# Transcriptomic analysis revealed the mechanism of oil dynamic accumulation during developing Siberian apricot (*Prunus sibirica* L.) seed kernels for the development of woody biodiesel

**DOI:** 10.1186/s13068-015-0213-3

**Published:** 2015-02-22

**Authors:** Jun Niu, Jiyong An, Libing Wang, Chengliang Fang, Denglong Ha, Chengyu Fu, Lin Qiu, Haiyan Yu, Haiyan Zhao, Xinyu Hou, Zheng Xiang, Sufan Zhou, Zhixiang Zhang, Xinyi Feng, Shanzhi Lin

**Affiliations:** College of Biological Sciences and Biotechnology, College of Nature Conservation, National Engineering Laboratory for Tree Breeding, Key Laboratory of Genetics and Breeding in Forest Trees and Ornamental Plants, Ministry of Education, Beijing Forestry University, Beijing, 10083 China; Research Institute of Forestry, Chinese Academy of Forestry, Beijing, 10091 China; Jigongshan National Nature Reserve, Xingyang, 464133 China; Liaocheng Food and Drug Administration, Liaocheng, 252000 Shandong China

**Keywords:** Siberian apricot, Woody biodiesel, Transcriptome sequencing, Differential expression, Oil accumulation mechanism

## Abstract

**Background:**

Siberian apricot (*Prunus sibirica* L.) has emerged as a novel potential source of biodiesel in China, but the molecular regulatory mechanism of oil accumulation in Siberian apricot seed kernels (SASK) is still unknown at present. To better develop SASK oil as woody biodiesel, it is essential to profile transcriptome and to identify the full repertoire of potential unigenes involved in the formation and accumulation of oil SASK during the different developing stages.

**Results:**

We firstly detected the temporal patterns for oil content and fatty acid (FA) compositions of SASK in 7 different developing stages. The best time for obtaining the high quality and quantity of SASK oil was characterized at 60 days after flowering (DAF), and the representative periods (10, 30, 50, 60, and 70 DAF) were selected for transcriptomic analysis. By Illumina/Solexa sequencings, approximately 65 million short reads (average length = 96 bp) were obtained, and then assembled into 124,070 unigenes by Trinity strategy (mean size = 829.62 bp). A total of 3,000, 2,781, 2,620, and 2,675 differentially expressed unigenes were identified at 30, 50, 60, and 70 DAF (10 DAF as the control) by DESeq method, respectively. The relationship between the unigene transcriptional profiles and the oil dynamic patterns in developing SASK was comparatively analyzed, and the specific unigenes encoding some known enzymes and transcription factors involved in acetyl-coenzyme A (acetyl-CoA) formation and oil accumulation were determined. Additionally, 5 key metabolic genes implicated in SASK oil accumulation were experimentally validated by quantitative real-time PCR (qRT-PCR). Our findings could help to construction of oil accumulated pathway and to elucidate the molecular regulatory mechanism of increased oil production in developing SASK.

**Conclusions:**

This is the first study of oil temporal patterns, transcriptome sequencings, and differential profiles in developing SASK. All our results will serve as the important foundation to further deeply explore the regulatory mechanism of SASK high-quality oil accumulation, and may also provide some reference for researching the woody biodiesel plants.

**Electronic supplementary material:**

The online version of this article (doi:10.1186/s13068-015-0213-3) contains supplementary material, which is available to authorized users.

## Background

With the increasing energy demand and environmental pollution problems caused by the fossil fuels, the renewable and clean of energy sources have become an inevitable choice for sustainable development of society and economy. Biodiesel, an alternative diesel fuel, was produced from biological sources such as plant oils or animal fats and has become a popular and environment-friendly fuel with renewability, low-emissions, security, and biodegradability [1]. However, the high cost of biodiesel has been the major barrier to its commercialization, and the application of edible oils to biodiesel production would lead to the problem of food versus fuel [2,3]. Recently, woody seed oils with a notable advantage over conventional feedstocks have been used as potential raw materials for biodiesel production [2,3]. Thus, it is important to develop woody resources for biodiesel. In our previous study, Siberian apricot has been characterized as a novel potential raw material for biodiesel from the ten candidate oleiferous tree species [3].

Siberian apricot (*Prunus sibirica* L.), a member of the family Rosaceae and the genus *Prunus*, is widely distributed in the temperate and mountainous districts of northern and northeastern China, eastern and southeastern regions of Mongolia, eastern Siberia regions, and maritime territory of Russia [4]. In China, Siberian apricot is one of the most economically and ecologically important and intensively studied tree species owing to its very plentiful resource, superior adaptability, and ecological benefits [5,6]. Moreover, the annual production of Siberian apricot seeds is above 192,500 tons and the average yield of seed oils is approximately 0.8 ton/ha in China [7]. Recently, the oils of SASK have been used in many fields, such as edible oils, lubricants, cosmetics, and surfactants, especially in biodiesel feedstocks [5,6,8]. Based on our evaluations of 17 selected Siberian apricot germplasms (oil content ranged from 44.73% to 57.83%) for biodiesel properties, the best Siberian apricot germplasm for biodiesel production has been identified with a high percentage of oleic (65.23% ± 4.97%) and linoleic acid (28.92% ± 4.62%), showing the potential for using SASK oil as a new source of biodiesel feedstocks in China [9-11]. Although considerable progress of Siberian apricot have been made in oil contents, FA compositions, biodiesel traits, ecological uses, afforestation, and medicinal utilizations [5,6,8-11], little is known about the molecular regulatory mechanism of oil accumulation in SASK. Thus, the transcriptomic analysis of developing SASK has become an imperative for the development of woody biodiesel.

In recent years, advances in low cost next-generation sequencing technology have made RNA sequencing (RNA-Seq) to become an effective choice for gene expression studies [12]. Although RNA-Seq has been performed on the transcriptomic analysis of many oilseed plants such as soybean, peanut, rapeseed, *Ricinus communis*, *Euonymus alatus*, *Landoltia punctata*, and *Tropaeolum majus*, and some known enzymes and genes were characterized to be involved in FA and triacylglycerol (TAG) biosynthesis [13-18], up to now reports about this situation only in oil palm, olive, and coconut for woody plants [19,20].

Recently, transcriptomic analysis for different tissues of Siberian apricot has been performed by 454 sequencing, and some candidate unigenes were annotated in oil biosynthesis [21], but the obtained results are not still suitable for us to deeply explore the biochemical and molecular mechanism of oil dynamic accumulation in developing SASK. In this work, the first kinetic patterns of oil contents and FA compositions were detected at different developing stages (10, 20, 30 40, 50, 60, and 70 days after flowering (DAF)), and the optimal periods for high quality and quantity of SASK oils and for comparative deep transcriptomic analysis were determined. The transcriptomes of developing SASK were sequenced by using Illumina technology, and then the assembled unigenes were functionally annotated. Moreover, the differentially expressed genes for the enzymes and transcription factors involved in oil accumulation of developing SASK were screened by DESeq method, and experimental validation of some key genes was performed by qRT-PCR. Overall, the temporal accumulated patterns of oils and FA compositions, and the transcriptional profiles of transcriptional regulatory factors and metabolic enzymes associated with the biosynthesis of pyruvate, acetyl-coenzyme A (acetyl-CoA), FA, TAG, and oil body were systematically analyzed in developing SASK, which will contribute to elucidate the molecular and metabolic mechanisms leading to increased oil biosynthesis and accumulation in SASK.

## Results and discussion

### Dynamic changes of oil content and FA compositions in developing SASK

**Figure 1:**
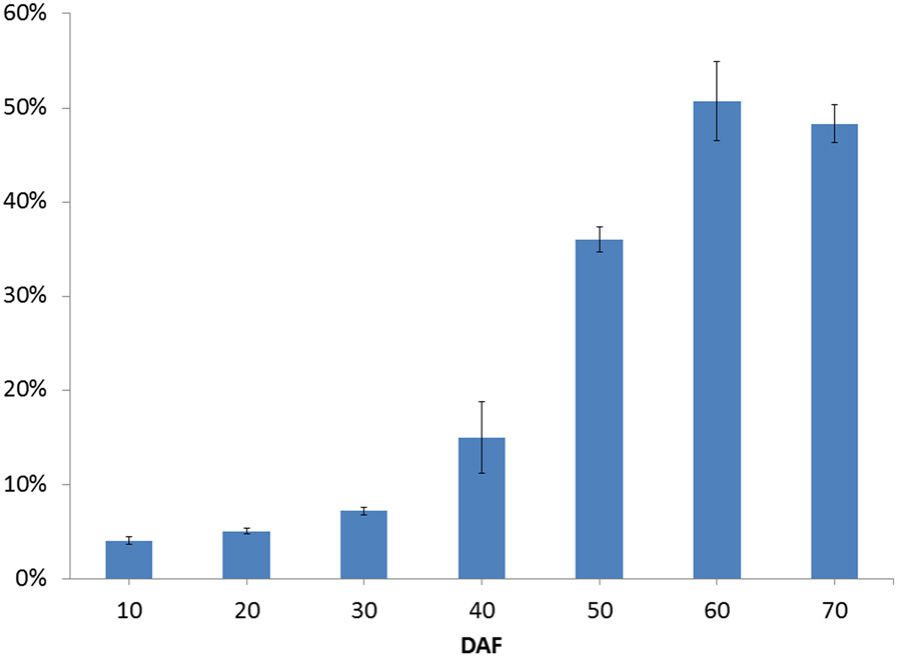
**The SASK oil content at different development period.**

**Figure 2:**
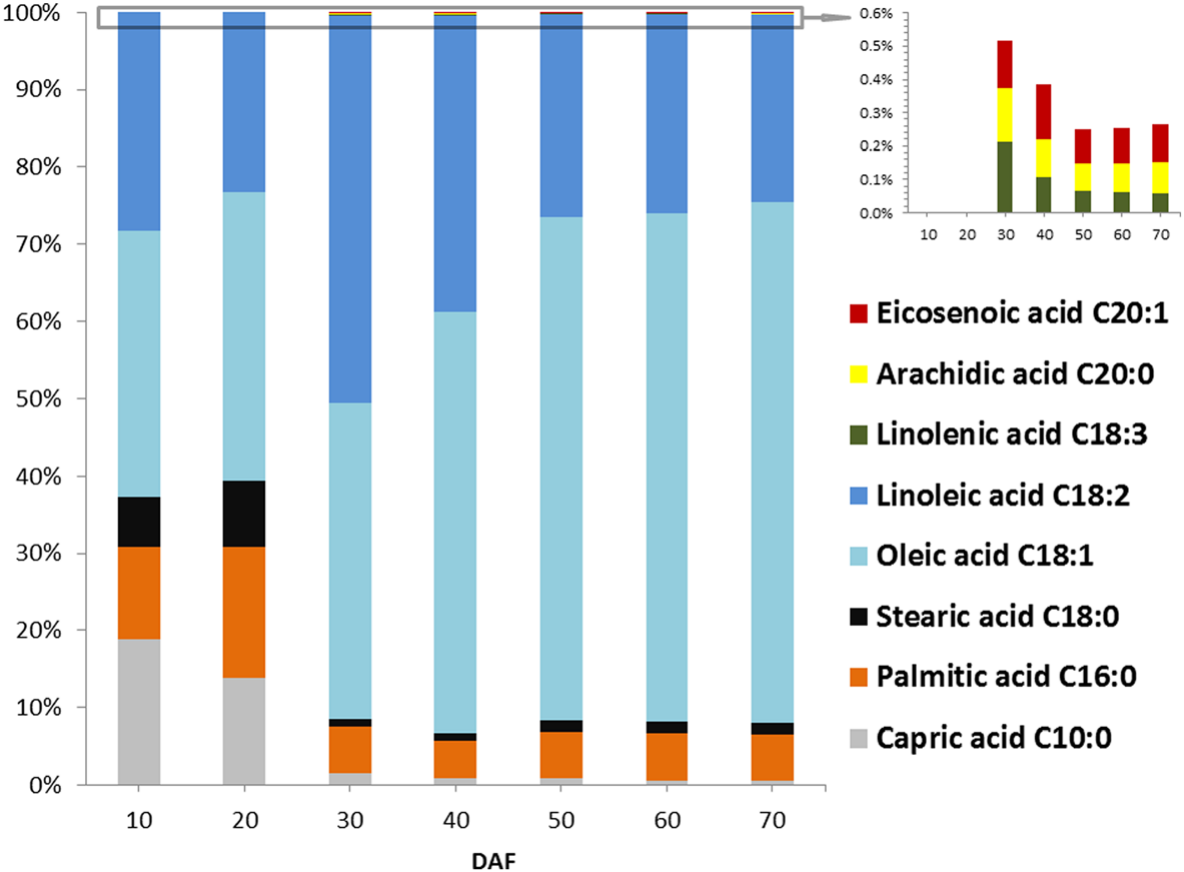
**Changes in the fatty acid composition during SASK development.** The data represent the mean of three independent measurements.

In general, high oil content is considered to be the vital metric for an energy-efficient plant. The study in different Siberian apricot germplasms has shown that the oil content of the fully ripened SASK, ranged from 44.73% to 57.83% [7,9,10], was higher than that of traditional oil plants such as *Jatropha curcas* (38.09%) and *Sapium sebiferum* (12% to 29%) [22], indicating a high commercial value for SASK oils. To explore the dynamic accumulated patterns of oils in developing SASK, we evaluated the SASK oil contents at different developing stages (Figure 1). There was a gradual increase in SASK oil content from 10 DAF (4.00% ± 0.39%) to 60 DAF (50.68% ± 4.18%), followed by approximately 2% decline at 70 DAF (fully ripe), indicating that the optimal harvest time for obtaining the maximum SASK oil content was at 60 DAF. It is important to note that the relative proportion of saturated, monounsaturated, and polyunsaturated FAs is the critical factor affecting biodiesel fuel properties [3]. In this study, the eight kinds of FAs were firstly detected in SASK oils at different developing stages, and the temporal patterns of their relative proportions were analyzed (Figure 2). We found that the C18:1 (oleic acid) relative proportion gradually increased from 10 DAF (34.37% ± 1.57%) to 70 DAF (67.41% ± 2.35%) with a remarkable elevated degree at 40 to 50 DAF, and the percentage of C18:2 (linoleic acid) exhibited a peak value (50.07% ± 2.87%) only at 30 DAF, but almost no significant alteration (23.29% to 38.45%) for the other different developing periods. Additionally, the other saturated or long chain FAs with a lower relative proportion showed a slight modification in developing SASK. Impressively, the total relative proportion of C18:1 and C18:2 in SASK oils ranged from 60.66% to 91.74% at 10 to 70 DAF (Figure 2), and therefore a higher ratio of oleic and linoleic acid revealed SASK oils with a high quality as a novel potential woody biodiesel feedstock in China, which corresponded to the previous investigations on SASK oils [7,10].

Taken together, our findings on the highest oil content (50.68% ± 4.18%) and the near-maximal total percentage of C18:1 and C18:2 (91.64%) in SASK at 60 DAF indicated that the very best harvest time for SASK oils with high quality and quantity is at 60 DAF. Moreover, according to the temporal patterns of oil content and the total relative proportion of C18:1 and C18:2 in developing SASK, the samples from five crucial periods (10, 30, 50, 60, and 70 DAF) were selected as the experimental materials for comparative deep transcriptomic analysis to better explore the molecular and metabolic regulatory mechanism of SASK oil increased-accumulation.

### Illumina sequencing and *de novo* assembly of developmental SASK

To clarify a global overview of the gene expressing profiles in developing SASK, a total of five cDNA libraries were constructed from different developing SASK RNA samples and were respectively sequenced by the Illumina transcriptome sequence. More than 5 GB raw data were respectively generated from 5 RNA-Seq samples, and then an average (55,994,330) of short reads were obtained after removing low-quality reads and adaptor sequences (Table 1). The numbers of raw data and short reads were larger in developing SASK than in longan [23] and peanut [15], suggesting that SASK Illumina sequencing could construct a complete read database. All high-quality reads were deposited in the NCBI Short Read Archive (SRA) database under accession numbers SRR1564517, SRR1568273, SRR1568275, SRR1568789, and SRR1568805.

**Table 1:** **Raw data and valid data statistics**

**Sample (DAF)**	**Raw data**		**Valid data**			**Valid ratio (reads) (%)**
	**Read**	**Base**	**Read**	**Base**	**Average length**	
10	60,940,126	6,094,012,600	53,830,008	5,182,918,614	96.28	88.33
30	65,980,058	6,598,005,800	58,413,976	5,636,764,333	96.50	88.53
50	64,572,540	6,457,254,000	56,984,496	5,479,436,388	96.16	88.25
60	72,078,976	7,207,897,600	57,262,494	5,368,833,289	93.76	79.44
70	62,898,984	6,289,898,400	53,480,678	5,112,244,068	95.59	85.03

**Figure 3:**
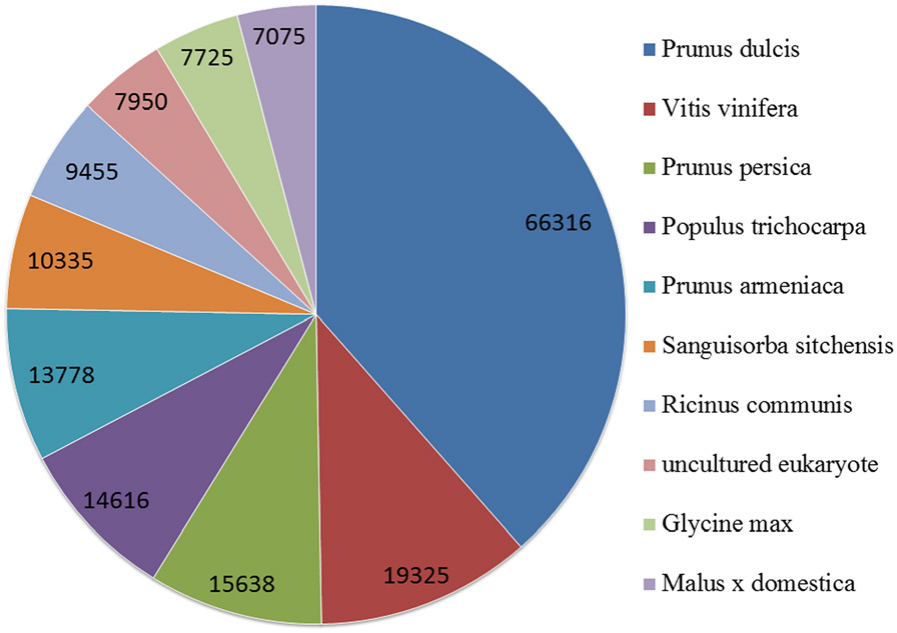
**Species distribution of the top BLAST hits for reads in Nr database.**

At present, there are no standard criteria to evaluate the quality of transcriptome assemblies [24]. To ensure accuracy and reliability of our SASK transcriptome assembly, 500,000 reads randomly selected from all reads were compared with NCBI database of nucleotide for pollution monitoring. From the Figure 3, the first *Prunus dulcis*, belonging to the same family Rosaceae with our experimental material (*P. sibirica* L.), was shown to have over three times as many reads as the second *Vitis vinifera*, indicating the reliability of our Illumina transcriptome sequencing data. Additionally, all these reads obtained from SASK of five developing stages were assembled by the Trinity software [25], and then low-complexity and low-quality reads were filtered out, resulting in 226,682 transcripts with GC content of 49.09%. By clustering through the Trinity program, we obtained 124,070 unigenes (N50: 1,603 bp) with the mean length of 829.62 bp (Additional file 1: Table S1) that is longer than those assembled from *Centella asiatica* (474 bp) [26], Yellow Horn (462 bp) [27], and peanut (751 bp) [15]. We also statistically found that there were 16,698 unigenes (13.46%) in the length range of 1,000 to 2,000 bp and 13,507 unigenes (10.88%) with length more than 2,000 bp (Additional file 2: Figure S1). Thus, a total of 30,205 unigenes (≥1,000 bp) in developing SASK, nearly 3 times more than our previous transcriptome analysis for different tissues of Siberian apricot [21], could massively fill and enrich the dataset of Siberian apricot.

### Functional annotation and classification of SASK unigenes

In order to understand specific functions of all the assembled unigenes in developing SASK, they were predicted via Basic Local Alignment Search Tool (BLAST) with a cut-off E-value of 10 to 5 in NCBI non-redundant protein (Nr) database, Swiss-Prot protein database (SwissProt), Arabidopsis proteome (AP) database, Clusters of Orthologous Groups (COG) database, Gene Ontology (GO), and Kyoto Encyclopedia of Genes and Genomes (KEGG). The BLAST results showed that 47,406 (38.21%), 30,511 (24.59%), 46,949 (37.84%), 17,870 (14.40%), 36,553 (29.46%), and 18,807 (15.10%) unigenes of developing SASK were highly similar with known proteins in Nr, SwissProt, AP, COG, GO, and KEGG databases, respectively (Additional file 3: Table S2 and Additional file 4: Table S3). Of all SASK unigenes, 49,904 (40.22%) homologous unigenes showed indeed that a considerable functional contig size was obtained. However, those unmatched SASK unigenes (74,166, 59.78%) might be as the putative tissue-specific novel genes or probably resulted from the shorter sequences with a lack of characterized protein domain to get BLAST hits [27].

The Gene Ontology (GO) annotation for the assembled unigenes was used to categorize the functions of the predicted SASK unigenes [28], and a total of 36,553 unigenes were assigned to the 3 main GO categories and 65 subcategories. Additional file 5: Figure S2 shows that ‘metabolic process,’ ‘cellular process,’ ‘binding,’ and ‘catalytic activity’ are the most dominant categories involving more than 20,000 unigenes, but only a few of genes were associated with terms such as ‘viral reproduction,’ ‘cell junction,’ and ‘channel regulator activity’. To further evaluate the completeness of our transcriptomic libraries and the effectiveness of the annotation processes, all SASK unigenes were aligned to the COG database to predict and classify by possible function [29]. The resulting 17,870 (14.4%) unigenes were assigned to the 25 COG classifications (Additional file 6: Figure S3), among which the cluster for ‘Posttranslational modification, protein turnover, chaperones’ represented the largest group (3,875, 21.68%) followed by ‘Signal transduction mechanisms’ (3,379, 18.91%) and ‘General function prediction only’ (2,662, 14.90%). Subsequently, to identify the biological functions and interactions of genes in developing SASK, the KEGG was applied to annotate the reference canonical pathways [30]. In total, 18,807 sequences were assigned to 313 KEGG pathways. Most of them were ‘Ribosome’ (1,254, 6.67%), ‘Protein processing in endoplasmic reticulum’ (604, 3.21%), ‘Oxidative phosphorylation’ (534, 2.84%), ‘Glycolysis/gluconeogenesis’ (494, 2.63%), and ‘Plant-pathogen interaction’ (490, 2.61%). Moreover, we also annotated several signaling networks of reproductive biology, such as ‘Non-homologous end-joining’ and ‘HIF-1 signaling pathway’.

### Analysis of differential gene expression for developing SASK

**Figure 4:**
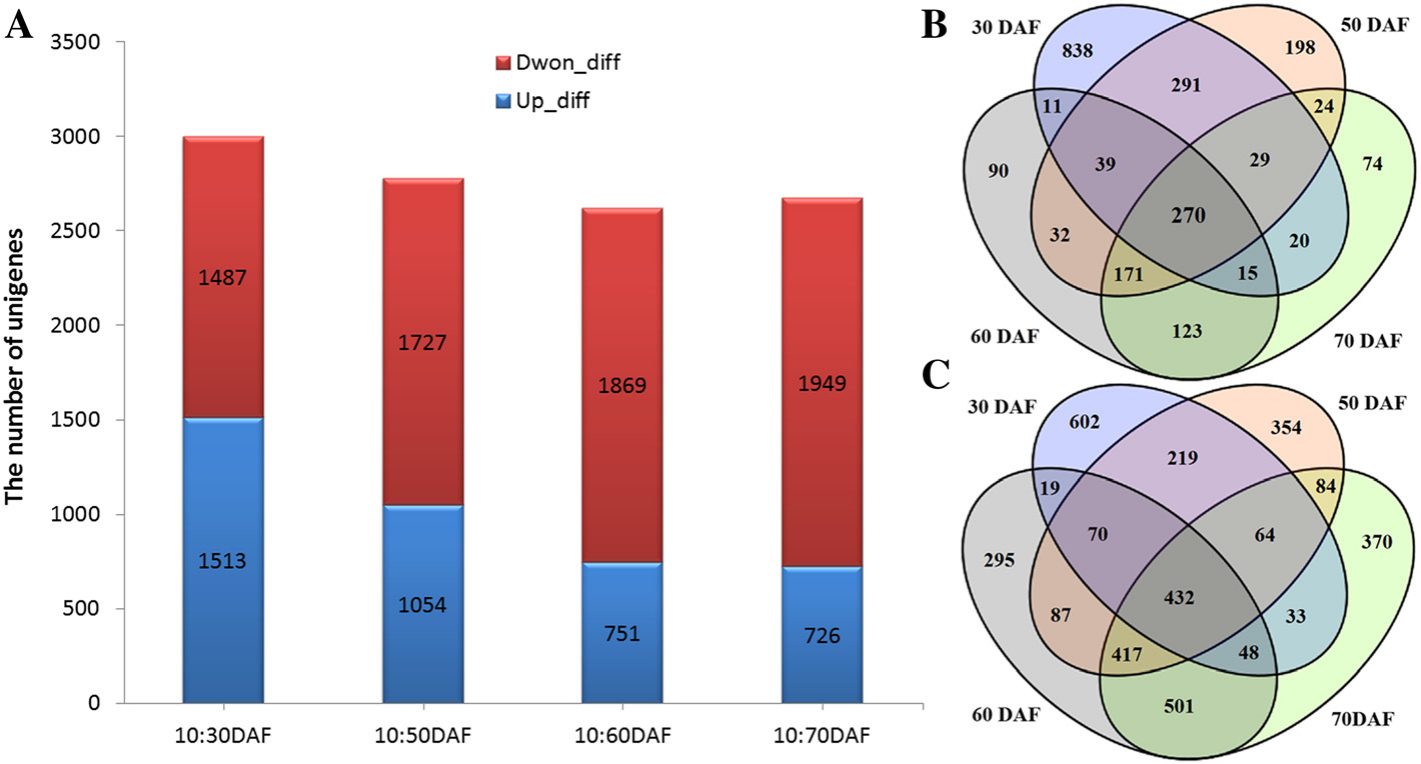
**Number and distribution of up-regulated and down-regulated genes. (A)** The number of up-regulated or down-regulated unigenes during developing SASK. **(B)** The distribution of up-regulated genes in differently developmental periods. **(C)** The distribution of down-regulated genes in differently developmental periods. The detailed sequences were showed in Additional file 9: Table S4.

To fully explore the differential expressions of the specific genes that are actually implicated in developing SASK, the clean reads from each library were mapped to the transcriptomic database for profiling the expressions of unigenes. The utilization rate of reads and the expression rate of unigenes showed that 10 DAF samples had the most unigenes (91,563, 74.28%) mapped to our constructed unigene database, whereas 50 DAF sample had the least unigenes (75,667, 60.9%) (Table 2 and Additional file 7: Figure S4). Also, the RPKM values were statistically calculated to select differential unigenes from developing SASK by using the DESeq method [31,32] (Additional file 8: Figure S5 and Additional file 9: Table S4). Our results revealed that 10:30 DAF exhibited the most up-regulated (1,513) and the least down-regulated unigenes (1,487), whereas 10:70 DAF had the least up-regulated (726) and the most down-regulated unigenes (1,949) (Figure 4A). By Venn diagram analysis, 838, 198, 74, and 90 up-regulated unigenes and 602, 354, 370, and 295 down-regulated unigenes were identified to be specific expression at 30, 50, 60, and 70 DAF (Figure 4B,C), respectively. Thus, combining with the temporal patterns of SASK oil at developing stages (Figure 1), we suggested that the candidate unigenes associated with oil accumulation pathway may be expressed at early stage of developing SASK.

**Table 2:** **Statistics of reads and the utilization of unigenes**

**DAF**	**Valid data**	**Map data**	**Data (%)**	**All unigene**	**Exp unigene**	**Unigene (%)**
10	53,830,008	49,899,224	92.70	124,070	92,163	74.28
30	58,413,976	54,258,107	92.89	124,070	84,912	68.44
50	56,984,496	53,171,111	93.31	124,070	76,668	61.79
60	57,262,494	51,322,910	89.63	124,070	75,667	60.99
70	53,480,678	47,986,764	89.73	124,070	84,030	67.73

### Transcript patterns for enzymes and transporters involved in generating pyruvate for SASK FA synthesis

**Figure 5:**
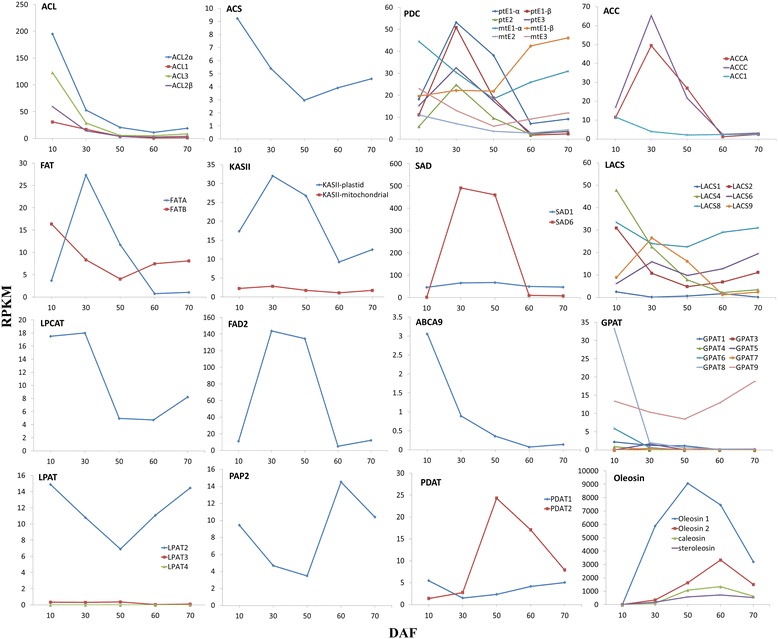
**Temporal profile of RPKM for genes encoding enzymes involved in the conversion of sucrose to TAG.** Abbreviations: PFK, phosphofructokinase; PFP, pyrophosphate-dependent phosphofructokinase; PK, pyruvate kinase; GLT, glycolipid transporter; TPT, triose phosphate transporter; GPT2, glucose-6-phosphate transporter; PPT1, phosphoenolpyruvate transporter; ACS, acetyl-CoA synthetase; PDC, pyruvate dehydrogenase complex; ACC, acetyl-CoA carboxylase; FAT, acyl-ACP thioesterase; KASII, ketoacylACP synthase II; SAD, stearoyl-ACP desaturase; LACS, long chain acyl-CoA synthetase; acyl-CoA: LPCAT, lysophosphatidylcholine acyltransferase; FAD2, fatty acid desaturase 2); acyl-CoA: glycerol-3-phosphate acyltransferase; LPAT, acyl-CoA: lyso-phosphatidic acid acyltransferase; PAP, phosphatidic acid phosphatase; PDCT, PC:DAG cholinephosphotransferase; and PDAT, phospholipid:DAG acyltransferase.

Sucrose is known as the most source of carbon, and its major metabolic flux through glycolytic pathway is to provide the large amounts of pyruvate required for *de novo* FA biosynthesis in oilseed plants [19,33,34]. In general, plant glycolysis occurs independently in two subcellular compartments of the cytosol and plastid. ATP-dependent phosphofructokinase (PFK) and pyruvate kinase (PK) are considered as two regulatory enzymes of glycolytic pathway in both cytosol and plastid, while pyrophosphate-dependent phosphofructokinase (PFP) only in cytosol has been characterized as a key enzyme to reversibly catalyze the conversion of fructose-6-phosphate to fructose-1,6-bisphosphate [16,19,35]. Moreover, the interchange of glycolytic intermediates between cytosol and plastid in plants has been implicated in the highly selective plastid transporters, including glycolipid transporter (GLT1), triose phosphate transporter (TPT), glucose-6-phosphate transporter (GPT2), and phosphoenolpyruvate transporter (PPT1) [19,33,36,37]. It is well known that the glycolytic substrates of hexose phosphates, generated from sucrose in the cytosol, are generally transported by GLT1 or GPT2 for plastidial glycolysis [34]. Recently, the transcripts of the plastid-localized enzymes (PFK and PK) and the transporters (GLT1 and PPT1) exhibited an obvious up-regulation, but PFP remained unchanged during ripening, indicating that plastid glycolysis plays a major role in providing pyruvate for high rates of FA synthesis in oil palm [34,35]. However, our transcriptional profiles revealed the stable-high expressions of cytosolic PFK (AT4G26270) and PK (AT3G52990) and the lower expressions of plastidial PFK (AT2G22480) and cytosolic PFP (AT1G12000) in developing SASK (Figure 5), suggesting a greater proportion of hexose to pyruvate flux in cytosol. Also consistent with this conclusion, the decreasing expressions in the translocators of GLT1 (AT2G33470), TPT (AT5G46110), and GPT2 (AT1G61800) were observed on developing SASK. It is, therefore, considered that there is a lower capacity to provide glycolytic substrates (hexose and hexose phosphate) and intermediate (triose phosphate) from cytosol to plastid in developing SASK. Interestingly, an up-regulated expression of the plastidial PK (AT5G52920) and its corresponding PPT1 (AT5G33320) were noted in SASK only at 30 DAF (Figure 5), the period that was important for initial FA biosynthesis (Figure 1). This implies a transportation of PEP from cytosol to plastid and the production of pyruvate required for SASK FA biosynthesis. Together, our results revealed that the cytosolic carbon supply via glycolytic pathway is crucial for *de novo* FA biosynthesis of developing SASK.

### Transcriptional patterns for the enzymes of acetyl-CoA formation for SASK *de novo* FA biosynthesis

Acetyl-CoA is an important precursor for *de novo* FA biosynthesis in plastids, various phytochemical biosynthesis and FA elongation in cytosol, and TCA cycle in mitochondria [38]. However, acetyl-CoA must be synthesized by specific synthetase in each subcellular compartment for a variety of important biosynthesis owing to the impermeability of biological membranes [39], indicating the multiple mechanisms of generating acetyl-CoA for different acetyl-CoA-requiring metabolisms. To our knowledge, acetyl-CoA synthetase (ACS) and pyruvate dehydrogenase complex (PDC) are thought to be implicated in the conversion of pyruvate to acetyl-CoA for *de novo* FA biosynthesis in plastids of plants, and four subunits (E1-α, E1-β, E2, and E3) of PDC have been characterized in Arabidopsis [16,39]. In an attempt to determine the importance of the enzymes (ACS and PDC) involved in acetyl-CoA formation for SASK *de novo* FA biosynthesis, their transcriptional patterns in developing SASK were analyzed in this study. The SASK homologies of Arabidopsis plastidial PDC E1-α (At1g01090), E1-β (At2g34590), E2 (At1g34430), and E3 (At4g16155) were identified as a significantly higher expression at 30 DAF, but the homologous gene of ACS (AT5G36880) displayed a lower expression (Figure 5), indicating that plastidial PDC may play an important role in the supply of acetyl-CoA for *de novo* FA synthesis in developing SASK. Attentively, PDC has also been characterized to locate in mitochondria of plants [40]. Indeed, our annotated four subunits of PDC in developing SASK exhibited high similarity with Arabidopsis mitochondrial E1-α (At1g59900), E1-β (At5g50850), E2 (At3g52200), and E3 (At1g48030), but their transcriptional patterns were shown no significant change (Figure 5), which could help to distinguish the PDC distribution between plastids and mitochondria in SASK at transcriptional level. Overall, the generation of acetyl-CoA might be tightly regulated in response of SASK to different developing stages.

### Identification of the *de novo* FA biosynthetic pathway-specific unigenes in developing SASK

*De novo* FA synthesis from acetyl-CoA precursors, as the first part of the lipogenesis process, is important in controlling the content and chain length (up to 18 carbons) of the saturated FAs in seed oils. Indeed, it has been reported that a multi-subunit acetyl-CoA carboxylase (ACC, EC: 6.4.1.2), regulated by phosphorylation and redox status as well as PII interactions, is the first committed key enzyme in the *de novo* FA biosynthesis pathway, and its physiological function is to provide the substrate malonyl-CoA for the biosynthetic saturated FAs [41,42]. Recently, it has been proposed that the intracellular accumulation of free C18:1, 18:1-CoA, and 18:1-acyl-carrier protein (ACP) in *Brassica napus* cell-suspension culture by feeding oleic acid (18:1) Tween esters could result in feedback inhibition of plastidial ACC activity, leading to a reduction of FA synthesis [43]. To characterize whether the transcriptional expressions of plastidial ACCs were linked to the FA biosynthesis in developing SASK, we performed a concurrent analysis of functional annotations and differential profiles for all the obtained unigenes. Here, a total of 10 SASK unigenes were characterized to be homologous with ACC subunits of other species, including 6 Arabidopsis ACC carboxyl transferase subunit alpha (ACCA), 2 Arabidopsis ACC1, and 2 *Populus trichocarpa* ACC biotin carboxylase subunit (ACCC), but only 2 homologies of *accA* and *accC* exhibited the highest expressing levels at 30 DAF followed by a dramatic decrease at 50 to 60 DAF (Figure 5). Combined with our observations on an obvious increase of FAs (C18:1 is one major component) at 50 DAF and the decreasing rate of FA biosynthesis after 50 DAF (Figures 1 and 2), we considered that a lower expressions of *acc* genes might be related with a high intracellular accumulation of FAs in developing SASK. Thus, our results prompt us to believe that an inhibitory expression of *acc* genes may be at 50 DAF, giving rise to a declined capacity of ACC to generate malonyl-CoA for *de novo* FA biosynthesis. However, further research is needed in transcriptional regulatory mechanism for plastidial *acc* as the target gene in *de novo* FA synthesis of developing SASK.

Before entering the synthesis pathway of FAs, malonyl-CoA from Acetyl-CoA is firstly transferred to malonyl-ACP by malonyl-CoA-ACP transferase (MAT, EC: 2.3.1.39) to provide two-carbon unit at each step of elongation. There are four reactions of condensation, reduction, and dehydration, and reduction occurred in each synthesized cycle, which is catalyzed by a series of regulatory enzymes. Our results of functional annotations revealed that one MAT, three 3-ketoacyl ACP synthase II (KASII, EC: 2.3.1.179), one 3-ketoacyl ACP synthase III (KASIII, EC: 2.3.1.180), two 3-ketoacyl ACP reductase (KAR, EC: 1.1.1.100), and one enoyl-ACP reductase (EAR, EC: 1.3.1.9) involved in FA preliminary synthesis in developing SASK. After seven elongated cycles, the production of saturated C16:0-ACP can either be converted to free C16:0 by the fatty acyl-ACP thioesterase B (FATB, EC:3.1.2.14 3.1.2.-) or further elongated by KASII to 18:0-ACP. Subsequently, 18:0-ACP can be hydrolyzed to free C18:0 by FATB or be desaturated to 18:1-ACP by 18:0-ACP desaturase (SAD, EC:1.14.19.2), and then 18:1-ACP is hydrolyzed to free C18:1 by the fatty acyl-ACP thioesterase A (FATA, EC:3.1.2.14 3.1.2.-) [44]. In plants, the relative proportion of FAs was remarkably altered by manipulation of any of these four enzymes (FATA, FATB, SAD, and KASII), but FATA and FATB have shown to be not directly involved in FAs biosynthesis [45,46]. In this study, the *fatB* genes with a stable expression were observed in developing SASK, while the *fatA* genes were notably up-regulated only at 30 DAF (Figure 5), suggesting that the resulting free C18:1 rather than C16:0 and C18:0 may be as one main product of plastid FA synthesis in developing SASK, which was coincide with our investigations on FA compositions (Figure 2). Additionally, a total of six *kasII* unigenes were found in our experiments, where 1 mitochondria *kasII* unigene with expressive stability and 5 plastid *kasII* unigenes with a high expression were identified at 30 DAF (Figure 5). It is interesting to note that 2 *sad6* genes were sharply up-regulated over 200 folds at 30 and 50 DAF, whereas 2 highly-expressed *sad1* genes remained stable in developing SASK (Figure 5). Thus, we considered that the increasing trend of C18:1 (oleic acid) content paralleled to the temporally transcriptional patterns for *sad6* and *kasII* in developing SASK (Figures 2 and 5) could explain SAD6 and KASII as the key enzymes for oleic acid formation in developing SASK. This also was strongly supported by the fact that a reduction in the activities of KASII and SAD caused by antisense gene silencing could lead to a decrease of 18:1 accumulation in Brassica and Arabidopsis seeds [47,48]. In conclusion, 18:1-ACP, as a major metabolite of FA biosynthesis, may be responsible for transportation of FA from plastid to cytosol in developing SASK.

### Determination of the unigenes-regulated polyunsaturated FA and TAG biosynthesis in developing SASK

The synthesized free FAs by the FATA/B thioesterases are exported from plastid to cytosol and then converted to the fatty acyl-CoAs by long-chain acyl CoA synthetase (LACS), which can provide the substrate for esterification in endoplasmic reticulum (ER) [49]. In this study, we observed a variety of kinetic transcriptional profiles for *lacs* in developing SASK. For example, *lacs4* and *lacs2* were up-regulated at 10 and 30 DAF, and two *lacs6* genes displayed high expression at 60 and 70 DAF, but *lacs8* showed stable expression during the whole developing periods, (Figure 5). It is concluded that multigene *lacs* genes may compensate for the lack of function each other in developing SASK, which could be evidenced by the fact that double (*lacs1* and *lacs9*) or triple (*lacs1, lacs8*, and *lacs9*) *lacs* mutant notably affect the FA content of Arabidopsis seeds [50].

For polyunsaturated FAs synthesis, C18:1 must be esterified by acyl-CoA: lysophosphatidylcholine acyltransferase (LPCAT) from cytosol to phosphatidylcholine (PC) pool in ER and then desaturated by omega-6 FA desaturase (FAD2) in PC pool [51]. In this work, four highly expressed *lpcat* genes at 10 to 30 DAF and two drastically up-regulated *fad2* genes at 30 to 50 DAF were detected in developing SASK (Figure 5), indicating that the polyunsaturated FAs may be as one of main components in SASK oil, which was also experimentally demonstrated on an accumulation of linolenic acid as the secondary main component in developing SASK (Figure 2). It is generally accepted that acyl-CoAs must be transported from the cytosol to ER for glycerolipid synthesis through diffusion by soluble carriers or by some more efficient inter-membrane transporters [44]. Recently, an ABC transporter (AtABCA9) has been identified to be involved in the delivery of FA to the ER in Arabidopsis [52]. However, SASK transcriptional profiles revealed that five unigenes encoding ABC transporter displayed a lower expression in developing SASK, of which two unigenes (comp63523_c1 and comp67480_c0) showed highly homologous with AtABCA9 transporter protein (Figure 5). Therefore, how this ABC transport system influence FAs transport in developing SASK remains to be further investigated.

The *de novo* formation of TAG occurs in ER via the Kennedy pathway, in which glycerol-3-phosphate (G3P) is initially acylated to produce TAG by the *sn-*G3P acyltransferase (GPAT, EC: 2.3.1.15) [53]. The previous studies of Arabidopsis GPATs showed that GPAT1 and GPAT4-8 with *sn*-2 regiospecificity were not required for TAG synthesis, and GPAT9 with *sn*-1 regiospecificity might be involved in TAG synthesis, but the function of GPAT2-3 with undetermined regioselectivity remained unknown [54,55], suggesting a functional divergence of the members in GPAT family. In this study, 2 genes of *gpat9* with a high and stable expression and 11 genes of *gpat1* and *gpat3*-7 with an ultralow expression were observed at different developing stages, but 2 genes of *gpat8* with specific up-regulation were only found at 10 DAF (Figure 5), indicating the difference of transcriptional expressions for the members of *gpat* multigene family in developing SASK. This investigation allowed us to speculate that the expressions of *gpat* multigene family may be tightly regulated in response to developing SASK. Moreover, together with the dynamic accumulated patterns of oils in developing SASK (Figure 1), we concluded that the GPAT9 may function as a key initial acyltransferase for SASK TAG assembly.

**Figure 6:**
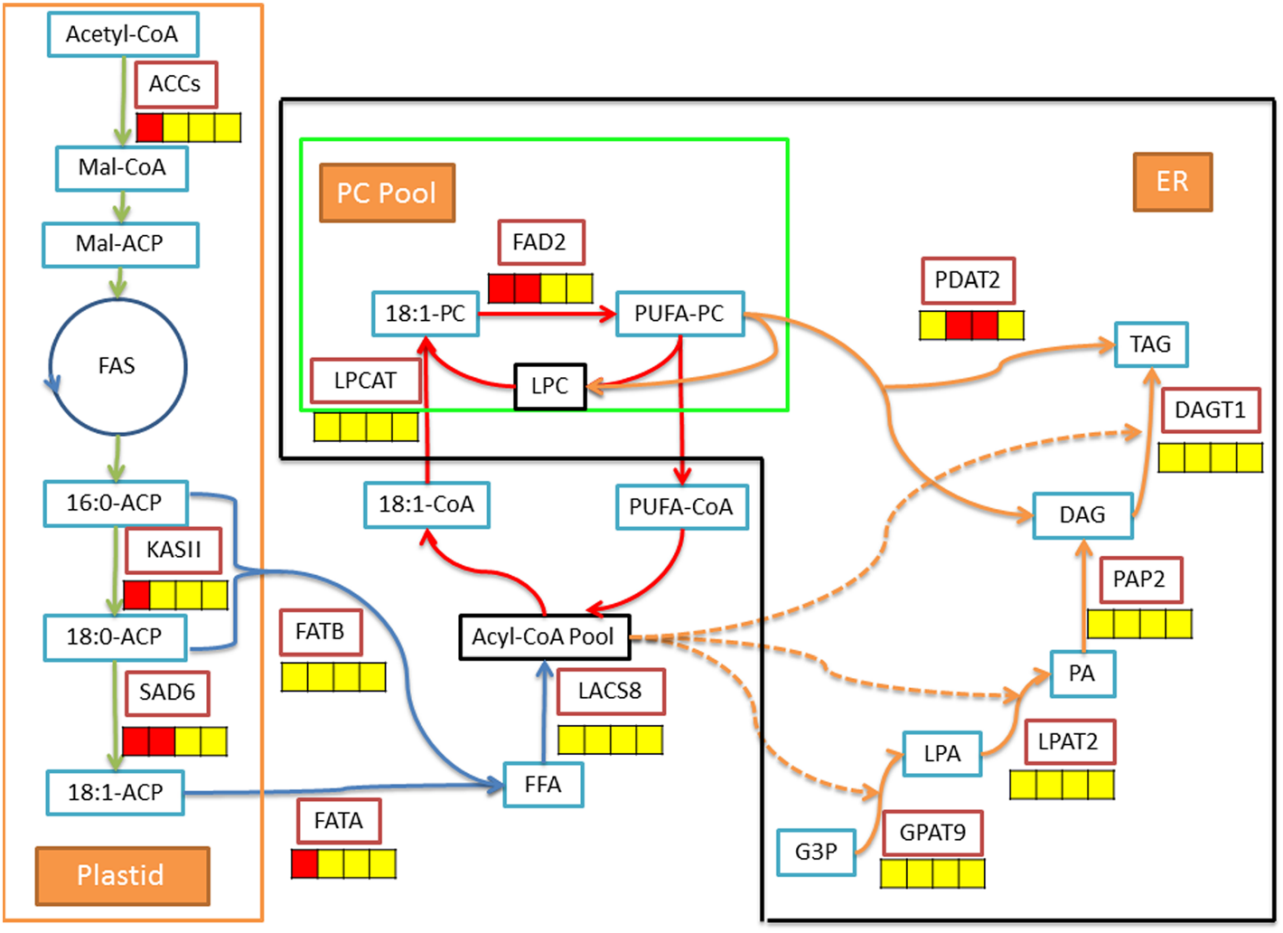
**The temporal patterns for enzymes involved in oil synthesis.** The icons below each enzyme show the result of DESeq analysis, from left to right: 10:30DAF, 10:50DAF, 10:60DAF, and 10:70DAF; red, up-regulation; yellow, no significant difference; green, down-regulation. Substrate abbreviations: ACP, acyl carrier protein; DAG, diacylglycerol; FFA, free fatty acid; G3P, glycerol-3-phosphate; LPA, lyso-phosphatidic acid; PA, phosphatidic acid; PC, phosphatidylcholine; PUFA, polyunsaturated fatty acids; TAG, triacylglycerol. Enzymatic abbreviations: ACC, acetyl-CoA carboxylase; DAG cholinephosphotransferase; DGAT1, acyl-CoA:DAG acyltransferase 1; FAD2, fatty acid desaturase 2; FATA/B, acyl-ACP thioesterase A/B; GPAT9, acyl-CoA:G3P acyltransferase 9; KASII, ketoacyl-ACP synthase II; LACS8, long chain acyl-CoA synthetase 8; LPAT2, acyl-CoA:LPA acyltransferase 2; LPCAT, acyl-CoA: lysophosphatidylcholine acyltransferase; PAP, PA phosphatase; PDAT2, phospholipid:DAG acyltransferase 2; PDCT: PC:DAG cholinephosphotransferase; SAD6, Stearoyl-ACP desaturase 6.

In the second step of *de novo* TAG assembly, lysophosphatidyl acyltransferase (LPAT, EC: 2.3.1.51) and phosphatidic acid phosphohydrolases (PAP, EC: 3.1.3.4) play the essential roles in the formation of phosphatidic acid (PA) and in the removal of the phosphate group from PA to form diacylglycerol (DAG), respectively. Also, several isozymes of LPAT and PAP have been demonstrated in higher plants [16,19,56-58]. Overexpression of rapeseed microsomal LPAT isozymic gene (*BAT1.13* or *BAT1.5*) could result in an increase in oil content of Arabidopsis seeds [56], but oil biosynthesis was not significantly affected by double knockout of two PAP genes (*PAH1* and *PAH2*) [57]. In order to assess the contribution of LPAT and PAP to DAG biosynthesis in developing SASK, the transcriptional patterns for the homologies of LPATs and PAPs were comparatively analyzed. Here, one LPAT homologous gene *lpat2* (AT3G57650) and four PAP homologous genes of *pah1* (At3g09560), *pah2* (AT5G42870), *lppδ* (AT3G58490), and *lpp3* (At3g02600) were characterized with an obvious increasing expression at 50 to 60 DAF (Figure 5), and therefore they are likely to be responsible for DAG biosynthesis in developing SASK. Recently, genetic analysis revealed that mutation of Arabidopsis *rod1* (encoding PC:DAG cholinephosphotransferase, PDCT) resulted in a marked decrease in polyunsaturated FAs (18:2 and 18:3) and a concomitant increase in 18:1 in seed, suggesting that PDCT may play an important role in the transfer of 18:1 into PC pool for desaturation and also in the reverse transfer of 18:2 and 18:3 into the TAG synthesis pathway in developing seeds [59]. In this work, the SASK homologies for PDCT in Arabidopsis (At3g15820) showed a much lower expression at 50 to 60 DAF (Figure 6). Thus, the contribution of PDCT to the accumulation of polyunsaturated FAs was very limited in developing SASK, which was in accordance with our detective results of FA compositions (Figure 2).

For the last step of the Kennedy pathway, multiple acyl-CoA:DAG acyltransferase (DGAT) enzymes have been demonstrated for the formation of TAG from DAG and Acyl-CoA in plants. For example, DGAT1, DGAT2, and DGAT3 are responsible for TAG production in Arabidopsis, tung tree, and peanut [60-62]. We annotated all the unigenes of developing SASK in this work. Surprisingly, only two *dgat1* genes were incrementally expressed in developing SASK, suggesting that DGAT1 might be one major enzyme specific to TAG biosynthesis of SASK. It was also noted that plant TAG synthesis could be catalyzed by phospholipid: diacylglycerol acyltransferase (PDAT) using the FAs from the PC pool [63]. We found two *pdat1* genes with a stable expression during the developing stages and two *pdat2* genes with a high expression at 50 to 60 DAF (Figure 5). Interestingly, the transcriptional level of *pdat2* was temporally paralleled to oil accumulation in developing SASK (Figure 1), implying that PDAT2 may play a major role in TAG synthesis of developing SASK.

Taken together, our investigations could help to characterize which isoforms of these large gene families are mainly responsible for SASK TAG biosynthesis, and also attested to the fact that Siberian apricot possesses many specific enzymes in TAG assembly (Figure 6), providing the important references for the study of lipogenesis process in woody oil trees.

### The transcriptionally temporal comparisons of oil body genes in developing SASK

TAGs can be stored as a form of oil bodies surrounded by a phospholipid monolayer and abundant amphipathic proteins such as oleosin, caleosin, and steroleosin in mature seeds [16,19,64]. By the transcriptionally temporal comparisons, we characterized the homologous oleosin-1 and oleosin-2 of Arabidopsis (AT4G25140 and AT5G40420)*,* caleosin of *Ficus pumila var* (ABV72237.1), and steroleosin of *Sesamum indicumm* (AAM46847.1) with an increasing or stable-high RPKM values in developing SASK (Figure 5). However, the RPKM for oleosin-1 was far higher than that for oleosin-2, steroleosin, or caleosin (Figure 5), which was very similar to the temporal patterns in developing oilseeds of *Brassica napus* [16].

### Detection of SASK transcription factors involved in oil synthesis

**Figure 7:**
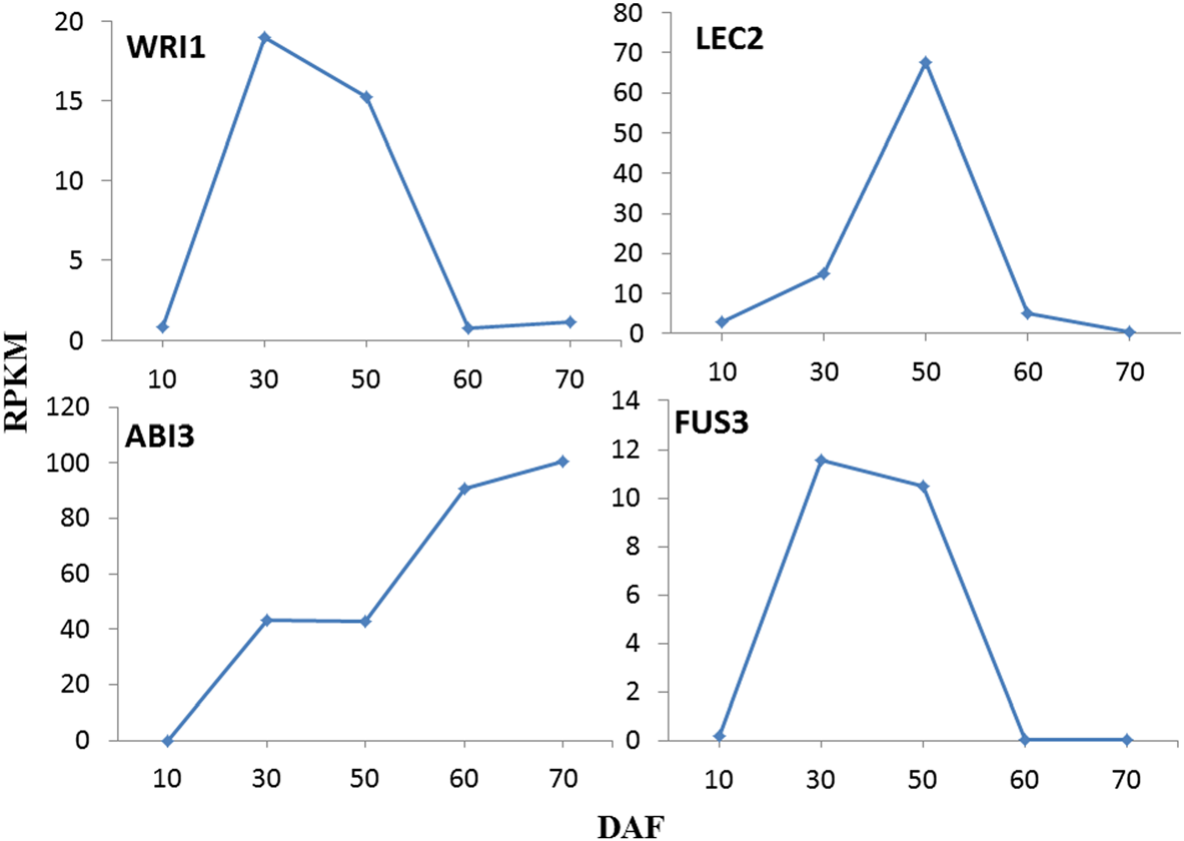
**Temporal pattern of RPKM for SASK transcription factors.**

In previous studies, the transcription factors (TFs) such as ABI3, LEC1, LEC2, FUS3, WRI1, PKL, FIE, or GL2 play the key roles in seed oil synthesis and deposition [65]. To gain an insight into transcriptional regulatory mechanism of SASK oil biosynthesis by TFs, we analyzed all the unigenes of developing SASK by BLASTX against Arabidopsis TFs. The resulting 25 unigenes at developing SASK were identified as 9 homologies of ABI3 (AT3G24650), WRI1 (AT3G54320), LEC2 (AT1G28300), FUS3 (AT3G26790), ADOF1 (AT1G51700), PKL (AT2G25170), AP2 (AT4G36920), GL2 (AT1G79840), and HSI2 (AT2G30470) in Arabidopsis, but none of these unigenes showed homology to LEC1, FIE, EMF2, or SWN (Additional file 10: Table S5). Moreover, our transcriptional profiling revealed that ABI3, WRI1, and LEC2 participated in positive regulation of the genes for oil synthesis in developing SASK, while AP2 and GL2 acted as negative regulatory factors. It is noteworthy that WRI1, the AP2-domain-containing transcription factor, is known to be up-regulated by LEC1, LEC2, ABI3, and FUS3 in Arabidopsis [66]. In our experiments, both FUS3 and WRI1 were shown with a high similar transcriptional profile in developing SASK (Figure 7), suggesting that the WRI1 gene might be a direct target of FUS3 in developing SASK. Additionally, the fact that plastidial *pk*, *ppt1*, *pdc*, and *acc* (*accA and accC*) displayed a coordinated transcriptional profile with *wri1* in developing SASK (Figures 5 and 7) indicated an involvement of WRI1 in the transcriptional regulation of target genes*,* as with Arabidopsis seeds [66]. Thus, SASK-WRI1 may play a contextual role in regulatory network of oil accumulation in developing SASK.

### Experimental validation and analysis of key genes involved in SASK oil accumulation

**Figure 8:**
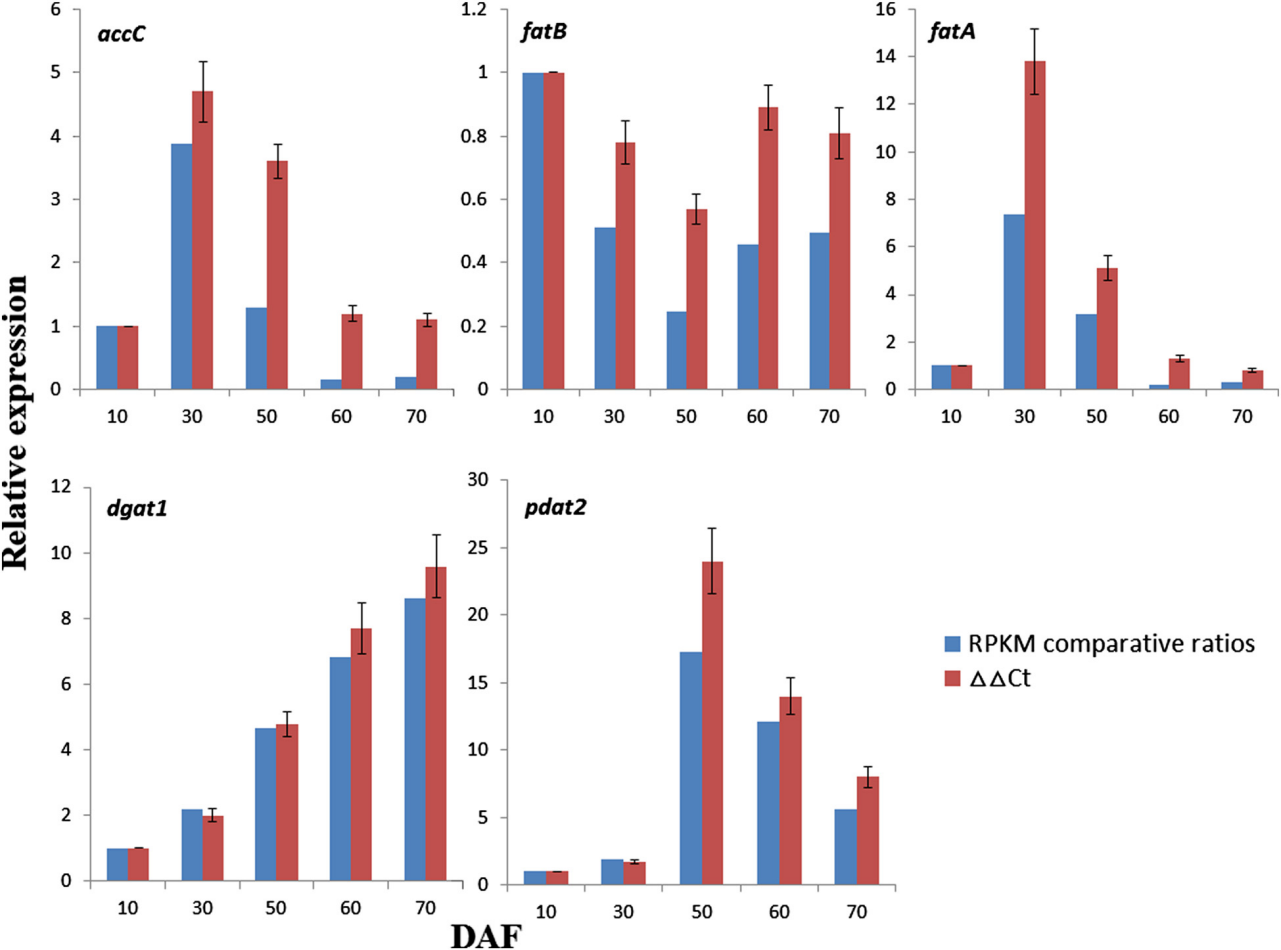
**Gene expression data obtained by qRT-PCR analysis.**

To validate RPKM data, the expression profiles for *accC*, *fatB*, *fatA*, *dgat1*, and *pdat2* related to oil biosynthesis in developing SASK were analyzed by using qRT-PCR (Table 3). Our results showed that the relative expression levels of these genes were mostly consistent with the RPKM comparative ratios (10 DAF as the control). However, only *dgat1* gene, not obtained further by DESeq method (Figure 6), was detected with significantly up-regulated expression in developing SASK by qRT-PCR analysis (Figure 8). Thus, differential expression analysis of SASK unigenes by DESeq method was reliable in this study.

**Table 3:** **Genes and primer sequences used for real-time PCR**

**Abbreviation**	**Gene name**	**Accession number**	**Primer forward**	**Primer reverse**	**Amplicon size (bp)**
*accC*	Acetyl-CoA carboxylase. biotin carboxylase subunit	KM364594	CCAGGAAGAATAACCGCCTAC	GGGAGTCATAGTTTGGAGGAAC	100
*fatB*	Fatty acyl-ACP thioesterase B	KM364595	GGCCCAAATATGCCAACAATC	GACGATCCATCCAGCCTTAAA	97
*fatA*	Fatty acyl-ACP thioesterase A	KM364596	TGATGTTCGGGAAGAGCATTTA	GGAGCAGGATCTTCCAGTTTAG	111
*dgat1*	Diacylglycerol acyltransferase 1	KM364597	CAGCCTATGTGTCTGCTTCTAT	CTCGATCCACACCTTGAGATT	98
*pdat2*	Phospholipid:diacylglycerol acyltransferase 2	KM364598	TGATGACAGTGTGCCTGTATTG	CTTGTGCTGGTACTCCCTTATG	115

It was hypothesized that the temporal transcriptional patterns for the genes involved in the biosynthesis of FAs and TAGs are very different in developing seed [16], which has been confirmed in our qRT-PCR results that *accC* and *fatA* for FA synthesis, and *dgat1* and *pdat2* for TAG formation in SASK were identified with a high expression at 10 to 30 DAF and 50 to 70 DAF, respectively (Figure 8). This appears to indicate the beginning synthesis of the FAs and TAGs before 30 DAF and after 50 DAF, respectively. It was reported that *dgat* or *pdat* mutation had minimal impact on oil content in Arabidopsis seeds, showing that the overlapping functions of PDAT and DGAT are essential for TAG biosynthesis [62]. Notably, our investigations on the maximum expressed value of *pdat2* and *dgat1* at 50 and 60 DAF respectively revealed an alteration of TAG accumulation in developing SASK by PDAT2 though PC pool or by DGAT1 via Kennedy pathway. All the above results evidenced that FAs and TAGs biosynthesis in developing SASK possessed complex dynamic regulation mechanisms, which deserve further research.

## Conclusions

The Siberian apricot, a potential woody oil species for biodiesel, has a high quality and quantity of oil and excellent adaptability to different growing conditions. To fully understand temporally accumulated patterns of oil content and FA compositions, seven samples from the whole developmental SASK were firstly detected and analyzed. As a result, 60 DAF were characterized as the best time to obtain the high quality and quantity of SASK oil, and 5 experimental samples from the representative periods (10, 30, 50, 60, and 70 DAF) were selected for comparative deep transcriptome analysis. Using the Illumina sequencing technology and Trinity program, 124,070 unigenes (mean length = 829.62 bp) were obtained from clean reads assembly and annotated in a public database, which could massively fill and enrich the dataset of Siberian apricot. There were 3,000, 2,781, 2,620, and 2,675 differentially expressed unigenes identified at 30, 50, 60, and 70 DAF using DESeq method (10 DAF as control), respectively. To reveal the accumulated mechanism of oil in developing SASK, the transcriptional profiles for the crucial transcription factors and metabolic enzymes involving in the biosynthesis of acetyl-CoA, FA, TAG, and oil body were systematically analyzed. We characterized that two transcription factors (WRI1 and FUS3) and some regulatory enzymes (PPT1, ptPK, ptPDC, ACC, KASII, SAD6, FATA, LACS8, FAD2, GPAT9, LPAT2, DGAT1, and PDAT2) play an important role in oil accumulation of developing SASK. Moreover, we also validated the temporal expression levels of five key genes (a*ccC, fatB, fatA, dgat1*, *and pdat2*) by qRT-PCR in developing SASK. All of these findings will be conducive to provide a substantial molecular biology background for researches on breeding, genetic diversity, and gene excavation of Siberian apricot and will promote the significant development for woody biodiesel. Certainly, we only analyzed a small subset of the mass data during SASK development and anticipate that other researchers would find valuable information from the SASK datasets available on request.

## Materials and methods

### Plant materials and extraction of SASK Oil

Siberian apricot is widely distributed in China, so it has not been listed as an endangered or protected species and does not require approval. The different developmental stages of SASK were obtained from the same tree located at the Beijing Forestry University experimental station, Beijing, China. The developmental processes of SASK from flowering to seed maturity were observed from May to July 2012. Flowers with the same anthesis were marked, and then seeds were harvested at 10 DAF (immature stage), 20 DAF, 30 DAF, 40 DAF, 50 DAF, 60 DAF, and 70 DAF (fully matured stage), respectively. After removing the sarcocarp, the seeds were immediately frozen in liquid nitrogen and stored at −80°C until use. The oil content of SASK and FA composition was determined using the previously described method [7].

### cDNA Library preparation and sequence data analysis and assembly

According to the detective results of SASK oil content and FA composition, the experimental SASK from the five crucial periods (10, 30, 50, 60, and 70 DAF) were selected as materials for comparative deep transcriptome and differential gene expression analysis. The equal weight of three biological samples from every developmental stage was mixed, and then total RNA was extracted from the mixture using RNeasy Plant Mini Kits (Qiagen, Inc., Valencia, CA, USA) according to the manufacturer’s protocol. Extracted RNA was qualified and quantified using a Nanodrop ND-1000 Spectrophotometer (Nanodrop Technologies, Wilmington, DE, USA), and all the samples showed a 260/280 nm ratio from 1.9 to 2.1; no sign of degradation was found. The samples for transcriptome analysis were prepared using Illumina’s kit following manufacturer’s protocol (Illumina, San Diego, CA, USA). Briefly, the poly-(A) mRNA was purified from the total RNA by Oligotex mRNA Mini Kit (Qiagen, Inc., Valencia, CA, USA) following the manufacturer’s protocol. cDNA library construction and normalization were performed using protocols described previously [67].

The cDNA library (two independent biological replicates) was sequenced on the Illumina sequencing platform (HiSeqTM 2000). After removal of the adapter sequences, the low-quality sequences (reads with ambiguous bases ‘N’) and reads with more than 10% Q < 20 bases, the quality of the clean reads were assessed by comparing the randomly selected 500,000 reads from all data to NT database with an E-value cut-off of 10^−10^ and coverage more than 80%. Subsequently, the clean reads were assembled into unigenes with the Trinity program [25].

### Sequence annotation

To understand their functions, the Siberian apricot unigenes were annotated using BLASTX alignment with an E-value cut-off of 10^−5^ against the following protein databases: NCBI nonredundant, Arabidopsis proteome, SwissProt, and eukaryotic orthologous groups. GO functional classifications and KEGG pathway assignments were performed, as was described previously and a detailed description of the workflow and settings used in the data analysis is given in [27].

### Differential expression analysis of unigenes

The unigenes expression levels were calculated using reads per kilobase transcriptome per million mapped reads (RPKM), which could eliminated the influence of gene length and sequencing level on the calculation of gene expression. The levels of unigenes expression in the different samples were compared using the DESeq method described in [31].

### qRT-PCR validation

Total RNA was extracted as the description for the cDNA library preparation and was reverse transcribed using the Reverse transcription System (Promega). The amplification primers were designed using PrimerQuest (http://www.idtdna.com/PrimerQuest/Home/Index) software with melting temperatures at 62°C, and the absence of secondary structures was verified by the UNAFold program (http://eu.idtdna.com/UNAFold) according to D’haene et al. [68]. According to our previous studies, cyclophilin gene and ubiquitin-conjugating enzyme gene were used as an internal control [69]. The qRT-PCR was performed using the SYBR Premix *Ex Taq* Kit (TaKaRa) according to the manufacturer’s protocol. Negative controls consisting of nuclease-free water instead of template, and reverse transcriptase controls prepared by substituting reverse transcriptase for nuclease-free water in the cDNA synthesis step were included in all analyses for each primer pair. Three technical repetitions were performed for qRT-PCR.

## Additional files

Additional file 1: Table S1. Summary of SASK sequencing and assembly. Additional file 2: Figure S1. Length distribution of the unigenes. Additional file 3: Table S2. All unigenes’ individual annotations, nt length, and levels of transcripts in RPKM. Additional file 4: Table S3. Detailed information of the annotated genes based on *Arabidopsis* proteome. Additional file 5: Figure S2. Histogram presentation of Gene Ontology classification. The results are summarized in three main categories: biological process, cellular component, and molecular function. The y-axis on the top indicates the number of genes, and the y-axis on the blow means the percent of genes in a category. Additional file 6: Figure S3. COG classification. A total of 17,870 unigenes were assigned to 25 classifications. The capital letters in x-axis indicates the COG categories as listed on the right of the histogram, and the y-axis indicates the number of unigenes. Additional file 7: Figure S4. RPKM distribution of unigene. Additional file 8: Figure S5. Comparative unigene expression profiles of five SASK developmental periods. Additional file 9: Table S4. All up- or down-regulated genes and function annotations in distinct developing SASK by DESeq analysis. Additional file 10: Table S5. The prediction of transcription factors involving in oil biosynthesis based on *Arabidopsis* protein.

## Abbreviations

acetyl-CoA: acetyl-coenzyme A
ACC: acetyl-CoA carboxylase
ACCA: ACC carboxyl transferase subunit alpha
ACCC: ACC biotin carboxylase subunit
ACP: acyl-carrier protein
ACS: acetyl-CoA synthetase
AP: Arabidopsis proteome
BLAST: Basic Local Alignment Search Tool
COG: clusters of orthologous groups
DAF: days after flowering
DAG: diacylglycerol
DGAT: acyl-CoA:DAG acyltransferase
EAR: enoyl-ACP reductase
ER: endoplasmic reticulum
FA: fatty acid
FAD2: omega-6 FA desaturase
FATA: fatty acyl-ACP thioesterase A
FATB: fatty acyl-ACP thioesterase B
GLT1: glycolipid transporter
GO: gene ontology
G3P: glycerol-3-phosphate
GPAT: *sn-1* G3P acyltransferase
GPT2: glucose-6-phosphate transporter
KAR: 3-ketoacyl ACP reductase
KASII: 3-ketoacyl ACP synthase II
KASIII: 3-ketoacyl ACP synthase II
KEGG: Kyoto Encyclopedia of Genes and Genomes
LPAT: lysophosphatidyl acyltransferase
LACS: long-chain acyl CoA synthetase
LPCAT: acyl-CoA:lysophosphatidylcholine acyltransferase
MAT: malonyl-CoA-ACP transacylase
Nr: NCBI non-redundant protein
PA: phosphatidic acid
PAP: PA phosphohydrolases
PC: phosphatidylcholine
PDC: pyruvate dehydrogenase complex
PDAT: phospholipid: diacylglycerol acyltransferase
PDCT: PC:DAG cholinephosphotransferase
PFK: ATP-dependent phosphofructokinase
PFP: pyrophosphate-dependent phosphofructokinase
PK: pyruvate kinase
PPT1: phosphoenolpyruvate transporter
qRT-PCR: quantitative real-time PCR
RNA-Seq: RNA sequencing
SAD: 18:0-ACP desaturase
SASK: Siberian apricot seed kernels
SRA: Short Read Archive
SwissProt: Swiss-Prot protein database
TAG: triacylglycerol
TCA cycle: tricarboxylic acid cycle
TPT: triose phosphate transporter
