# Supplementary material for: Transcriptomic analysis revealed the mechanism of oil dynamic accumulation during developing Siberian apricot (Prunus sibirica L.) seed kernels for the development of woody biodiesel
Source: Biotechnol Biofuels. 2015 Feb 22;8:29. doi: 10.1186/s13068-015-0213-3 (PMC4381669; doi:10.1186/s13068-015-0213-3)
Supplement: Additional file 1: Table S1. — Summary of SASK sequencing and assembly. [file 13068_2015_213_MOESM1_ESM.docx]

**Table S1**

Summary of SASK sequencing and assembly

|  | **All (>=200bp)** | **>=500 bp** | **>=1000 bp** | **N50** | **N90** | **Total Length** | **Max Length** | **Min Length** | **Average Length** |
| --- | --- | --- | --- | --- | --- | --- | --- | --- | --- |
| **Transcript** | 226682 | 140430 | 100281 | 2176 | 535 | 288188440 | 15503 | 201 | 1271.33 |
| **Unigene** | 124072 | 52631 | 30205 | 1603 | 305 | 102932105 | 15503 | 201 | 829.62 |
