# Supplementary figures and images for: Transcriptomic analysis revealed the mechanism of oil dynamic accumulation during developing Siberian apricot (Prunus sibirica L.) seed kernels for the development of woody biodiesel
Source: Biotechnol Biofuels. 2015 Feb 22;8:29. doi: 10.1186/s13068-015-0213-3 (PMC4381669; doi:10.1186/s13068-015-0213-3)

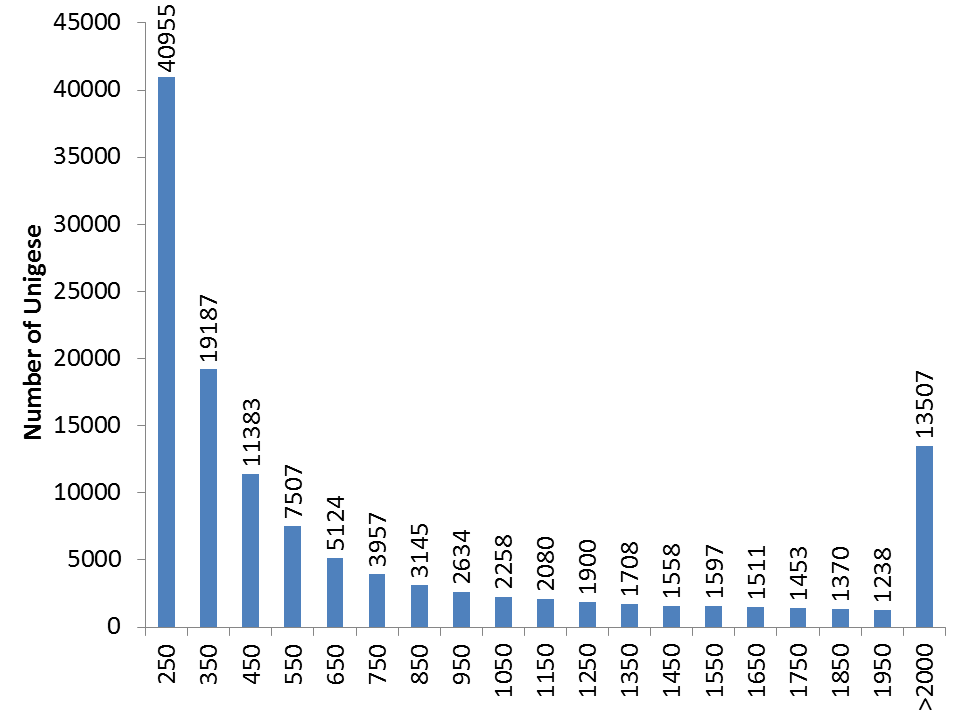

Supplement: Additional file 2: Figure S1. — Length distribution of the unigenes. [file 13068_2015_213_MOESM2_ESM.tiff]

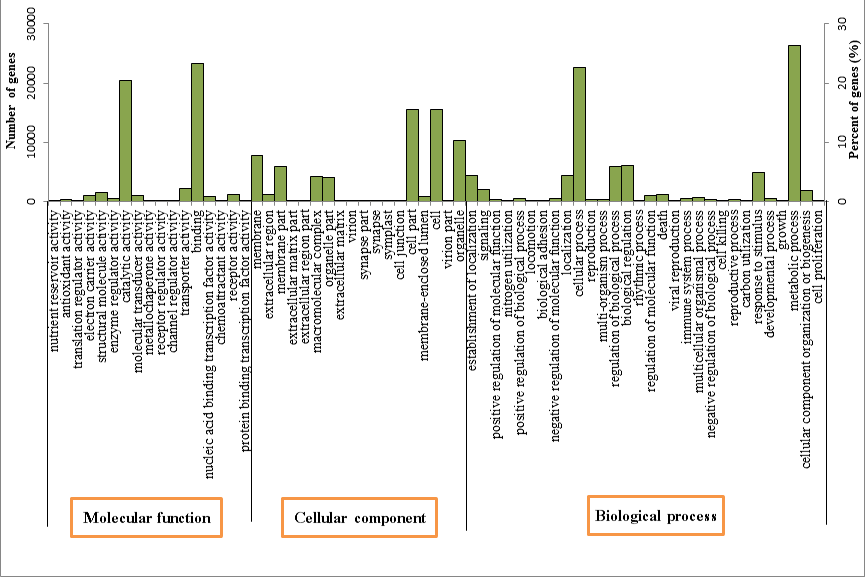

Supplement: Additional file 5: Figure S2. — Histogram presentation of Gene Ontology classification. The results are summarized in three main categories: biological process, cellular component, and molecular function. The y-axis on the top indicates the number of genes, and the y-axis on the blow means the percent of genes in a category. [file 13068_2015_213_MOESM5_ESM.tiff]

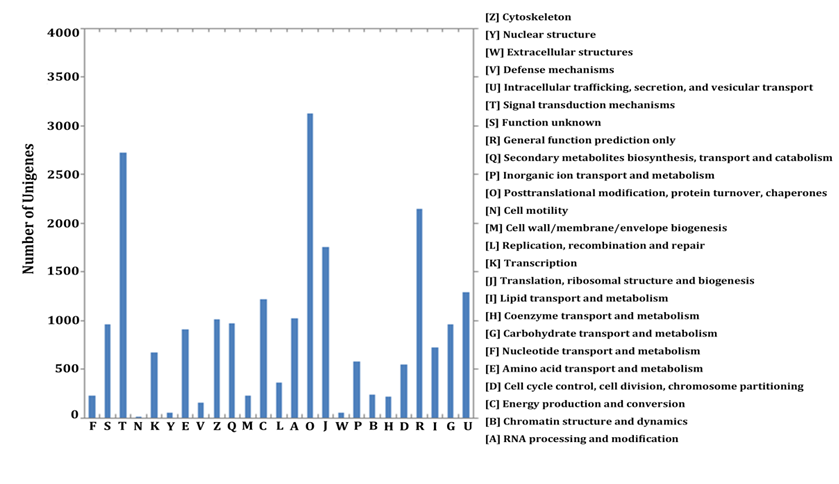

Supplement: Additional file 6: Figure S3. — COG classification. A total of 17,870 unigenes were assigned to 25 classifications. The capital letters in x-axis indicates the COG categories as listed on the right of the histogram, and the y-axis indicates the number of unigenes. [file 13068_2015_213_MOESM6_ESM.tiff]

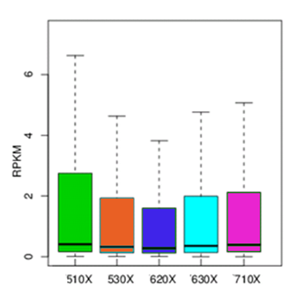

Supplement: Additional file 7: Figure S4. — RPKM distribution of unigene. [file 13068_2015_213_MOESM7_ESM.tiff]

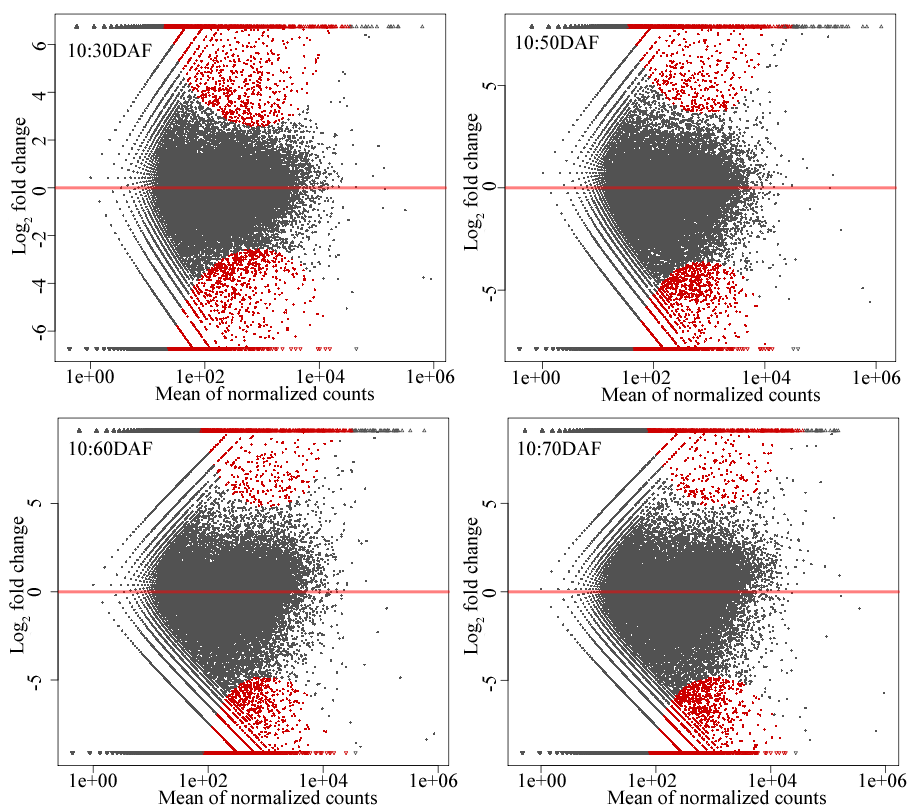

Supplement: Additional file 8: Figure S5. — Comparative unigene expression profiles of five SASK developmental periods. [file 13068_2015_213_MOESM8_ESM.tiff]
